# Consanguinity: Still a challenge

**DOI:** 10.4103/0019-5545.44897

**Published:** 2009

**Authors:** T. S. Sathyanarayana Rao, M. R. Asha, K. Sambamurthy, K.S. Jagannatha Rao

**Affiliations:** Department of Psychiatry, JSS University, JSS Medical College Hospital, Ramanuja Road, Mysore - 570 004, India; 1 Reiki Practitioner and Freelance writer, 788/160, 18^th^ Cross, Ramanuja Road, Mysore, India; 2 Department of Neuroscience, Medical university of South Carolina, Charleston, SC, 29425, USA; 3 Biochemistry and Nutrition, CFTRI, Mysore-570020, India

We are all connected to life. Every choice we make and every belief we hold exerts influence upon the whole of life. And we live with the consequences of our choice. As part of our biological health, this unique truth has physical expressions in honor, loyalty, family and group bonds. Probably this forms the basis of marriage, one of the most vital and powerful of our relationships. The human population has seen modern civilization and is still within family boundaries. One such familial-social bond in consanguineous marriage.

The word consanguineous comes from the two Latin words “con” meaning shared and “sanguis” meaning blood. Consanguinity describes a relationship between two people who share an ancestor, or share blood. Such marriages are favoured by different populations usually bound to traditional customs, beliefs and to keep property in united form within the family. In Arab Muslim communities, first cousin unions between a man and his father's brother's daughter are preferred. However, in population of Dravidian Hindus of South India, marriage of a boy with his mother's brother's daughter, is opposed. But, uncle-niece unions (but not aunt-nephew) are permitted in Judaism. Many studies indicated that consanguineous marriages are strongly favoured in human populations. The highest consanguineous marriages (20% to over 50%) are reported in North of Africa, Asia etc, usually associated with low socioeconomic status, illiteracy, and rural residence.[1,2] In India, the main reasons for these marriages are stronger family ties, the integrity of estates and the like. But the current debate in medical sciences is on the health implications of these consanguineous marriages.

Consanguineous marriages are major responsible risk factors for Bipolar disorders. This marriage system has been reported as an important factor in the appearance of autosomal recessive diseases and congenital anomalies, including hydrocephalus, postaxial hand polydactyly and bilateral cleft lip cleft palate, bipolar disorders, depression, dysferlinopathy, reproductive disorders, sterility, infant mortality, child deaths, spontaneous abortions and stillbirths etc. Also there are reports indicating positive association between consanguinity and Down syndrome, and also ventricular septal defect (VSD), atrial septal defect (ASD), atrioventricular septal defect (AVSD), pulmonary stenosis (PS) and pulmonary atresia (PA). The risk for birth defects in the offspring of first cousin matings has been increased to 5-8% compared to 2-3% in non-consanguineous marriages.[3] However, first cousin unions are culturally preferred. The reason is ease of marriage decision making when the potential spouse is well-known and considered to be part of the ‘extended family’. And these marriages also tend to reinforce social and kin bonds from one to the next generation.

Recently Nalini and Gayathri[4] reported the role of Consanguinity (46.4%) in causing Dysferlinopathy in 28 patients. Further Bindu *et al*,[5] reported the role of Consanguinity (61.5%) as a etiological factor for Hallervorden-Spatz syndrome (HSS), a rare autosomal recessive neurodegenerative disorder of childhood. Reports from India and west proved beyond doubt that consanguinity plays a significant role in mental health problems. All these disorders play a significant role on world economy and productivity and become a huge burden on medical fraternity. In spite of medical advancements, literacy rate and urbanization, still this family linked traditions are not able to be broken. In recent times, the situation appears better in urban areas. In a population with a high degree of inbreeding, the formulation of a public health program with multi-approach strategy, including education about the anticipated genetic consequences, prenatal diagnosis, neonatal screening, and genetic counselling, is a necessity.

## GENETIC COUNSELING

Redefining conventional medical ethics in the light of new moral issues arising out of advances in medical science and technology has resulted in the emergence of Applied Ethics.

Genetic counseling is the process by which clients or their relatives, at risk of an inherited disorder, are advised of the consequences and nature of the disorder, the probabilities of developing or transmitting it, and the choices open to them in management and planning of their families, in an attempt to prevent, avoid or ameliorate the disorder. This has preventive, diagnostic, therapeutic and supportive value. Genetic counselors function as members of health care team and act as patient advocates, protecting their best interests in addition to working as a genetic resource to physicians. Genetic counselors provide useful information and support to families who may be at risk of an array of inherited disorders. They are involved in identification of families at risk, investigation of problems presented by the family, interpretation of information about the disorder, analysis of inheritance patterns and evaluation of risks of recurrence while reviewing testing options available to the family.

It is important to look for inborn errors of metabolism in children of consanguineous parents, since many of these conditions are inherited in an autosomal recessive manner. While individually sparse, collectively they represent a significant burden of disease. Nevertheless, a ray of hope here is, some conditions are treatable if diagnosed at an early stage. Consanguineous couples with a child having an undiagnosed medical condition are at a risk of higher chance than their unrelated counterparts, of future children being affected, due to the possibility of an unrecognized autosomal recessive condition.

When an abnormality or illness is identified in a child of a consanguineous couple, it is imperative that investigations and referrals should proceed in a systematic way, as clinically indicated for the presenting symptoms but with an emphasis on autosomal recessive conditions in the diagnosis. Nevertheless, it is important to remember that autosomal recessive conditions can arise by chance, with the child having two different mutations, not necessarily because of consanguineous marriage alone. This can go a long way in eliminating shame and guilt in the parents, that, somehow they were responsible for the condition of their child or in erasing the misconception that they are being punished by God for their sin of marrying within the close family.

Apart from autosomal recessive conditions leading to learning difficulties, consanguinity has not been reported to have any significant effect on intelligence.

Presence of family history of possible autosomal recessive condition may considerably increase the risk of offspring over the background risks of consanguinity. In such cases, the consanguineous couple can be tested for their carrier status and prenatal diagnosis when necessary. When the diagnosis or mutation is not known, investigation of an affected relative may provide valuable clues. If this is not possible, an estimation of risks involved and detailed, systematic fetal scans are the only choice open. However, this may leave many couples disconcerted at the residual uncertainty.

In the case of death of the affected child, or termination of affected pregnancy, a postmortem exam may throw light on the causal factors. This presents the best chance under the circumstances to make the diagnosis and identify the causal mutation. While being distressful to the grieving parents, this examination may reveal information that may enable their doctors to predict the health status of the next child/children. It is important to remember that, it could be extremely frustrating for a couple if, in a future pregnancy, the lack of relevant genetic information about their first child makes it impossible to provide accurate advice and testing.

The general risk for any couple of begetting a child with a serious or lethal medical condition is around 2%. The higher risk for a couple who are related as first cousins, in the absence of a known genetic disease in the family, is 3%. This statistical estimation often comes as a relief for the genetic counselees who anticipate a significantly higher figure. This is one of the benefits of research in this field that provides authentic data based on which probabilities, close to reality could be predicted. The higher risk comes as a consequence of autosomal recessive conditions stemming from homozygosity by descent. In other words, it is the risk of a recessive mutation present in an ancestor being passed down two branches of the family and coming together in the consanginous marriage. It is assumed that all of us carry at least one mutated allele that would lead to an autosomal recessive condition if present in two copies (homozygosity). In case this mutant allele is inherited by both members of a consanguineous marriage from a common ancestor, they both will be carriers for this condition. Hence they will have a one in four chance of begetting an affected offspring.

The possibility of both parents being carriers for a recessive condition is influenced by how closely they are related. This means that the offspring risk can be minimized while retaining the social and familial advantages of consanguinity, if weddings are consummated between distant relatives (third cousins rather than second cousins or second cousins rather than first cousins).

An interesting angle is lent to this scenario by “Astrology”, an ancient practice prevalent in many communities the world over, especially in India. “Horoscope matching” to check “Marital compatibility” of the boy and girl before wedding, which is religiously and meticulously followed by elders on both the sides, may be viewed as an intelligent form of “Premarital consultation / counseling”.

Genetic counseling yields best results when done premaritally or at least prior to conception. A non-judgmental attitude towards consanginous couples is essential on the part of the counselor, to establish good communication channels and to foster effective working relationships between the medical profession and communities where consanguineous marriages are prevalent.

Some useful tips in counseling consanguineous couples:

1. Refer well before conception occurs especially if they have a family history of a possible autosomal recessive condition.
2. Remember to empathize and not to imply that a child's condition is the parents' fault, even if the couple are consanginous and the child has two identical copies of a mutant gene. Nobody chooses to deliberately pass on an illness to their offspring and no one is to blame.
3. Ensure that the couple referred for premarital genetic counseling are made aware that there are no blood tests available that provide “General Genetic Compatibility” data. They need to be informed that some few basic carrier tests are there for a limited range of specific conditions.
4. Adopt a non-judgmental attitude with a positive mindset to disseminate knowledge and information to the couple, empowering them with the various options available, enabling them to make intelligent decisions.
5. Deal with the issue in a sensitive, caring and sensible manner.

The young age of marriage in consanguineous couples further implicates a need to increase awareness programs among the young generation about the deleterious effects of consanguineous marriages. It is clear that the social benefits derived from such marriages are of paramount importance to consanguineous couples; however, the availability of preventive measures should be emphasized. Further genetic investigation conducted in this area to elucidate the mode of inheritance is required.[6]

India needs to take a big leap in this direction with consanguineous marriages being more prevalent. The need of the hour is setting up infrastructure with basic research and good medical facilities with genetic testing and counselling. Many hospitals in our country lack genetic testing facilities with few well trained genetic counsellors to handle the situation. Adopting better translational research concept and intervention strategies help consanguineous couples reach informed and intelligent reproductive decisions, with which they have to live throughout their lives.
